# Features of cognitive functions in generalized anxiety disorder: narrative review

**DOI:** 10.1192/j.eurpsy.2024.879

**Published:** 2024-08-27

**Authors:** E. L. Isakulyan, M. P. Marachev

**Affiliations:** ^1^ Neurocenter of medical and psychological correction and rehabilitation; ^2^Moscow Scientific Research Institute of Psychiatry Branch of V. Serbsky National Medical Research Centre for Psychiatry and Narcology, Moscow, Russian Federation

## Abstract

**Introduction:**

Generalized anxiety disorder (GAD) is characterized by excessive and uncontrollable worry and anxiety about several activities or events. Although some cognitive symptoms are common in GAD patients, there are still controversial results from their linkage. Some studies indicate intact cognitive functions in GAD patients, while others suggest that anxiety and its cognitive aspect, worry, are associated with reduced performance in several cognitive domains.

**Objectives:**

To assess the linkage and contribution of cognitive impairment to the maintenance and severity of GAD; to determine which specific domains of cognitive function are impaired in patients with GAD; and to examine age differences regarding cognitive impairment in GAD patients

**Methods:**

A systematic literature search was executed using the PubMed and Google Scholar databases from 1960 to 2023 and the keywords “generalized anxiety disorder”, “anxiety disorder” “cognitive function”, “cognitive dysfunction”, “cognitive impairment”, “late-life”, “young”, “adult”, and their combination.

**Results:**

Anxiety and worry, as main characteristics of GAD, were shown to be linked and manifested by deficient attentional control, a main function of working memory. Attentional control functions are biased toward threats, which, in turn, hinders cognitive processing efficiency. Moreover, several structural and electrophysiological impairments could be linked to cognitive dysfunction in people with GAD. For example, patients with GAD showed reductions in gray matter volumes, especially in the regions of the hip, midbrain, thalamus, insula and superior temporal gyrus. The hippocampus, middle cingulate gyrus, putamen and head of the caudate nucleus also showed lower activity in response to the neutral words. Also, GAD patients have better inhibitory control, which may be associated with more severe symptomatology. These results are consistent with attentional control theory, which posits that worry might negatively impinge on inhibition and set-shifting. In terms of age differences, we observed that GAD in elderly patients is associated with impairment of short-term and delayed memory. In young adults, GAD is associated with various cognitive impairments, particularly in selective attention, working memory, cognitive flexibility, planning ability or efficiency, and other executive functions (EF).

**Conclusions:**

To sum up, we observed that GAD is associated with worse cognitive functioning in several domains. Further research into cognitive dysfunction in GAD is needed to better understand the impact on daily living and to allow more tailored treatment strategies including medication, therapy and interventions targeted to improve specific cognitive domains.

**Disclosure of Interest:**

None Declared

